# A river (of liquid metal) runs through it

**DOI:** 10.1093/nsr/nwaa018

**Published:** 2020-02-10

**Authors:** Michael D Dickey

**Affiliations:** Department of Chemical and Biomolecular Engineering, North Carolina State University, USA

Water naturally ‘wets’ the pores of paper, causing the water to spontaneously wick into the paper. However, liquids do not wick into all porous materials. For example, pores in raincoats let heat pass from your body to the surroundings, yet water does not penetrate into the pores. The extent to which liquids pass through pores depends on their contact angle with the material (water on a waxed car has a high contact angle) and the surface tension of the liquid. Water, which has a large surface tension, will only pass through pores that it wets. Liquid metals have surface tensions that are nearly an order of magnitude larger than water and wet very few surfaces. As a result, they tend to assume a spherical shape and do not like to pass into porous surfaces. In fact, there is a technique called ‘porosimetry’ that determines the pore size of a material by measuring the pressure required to physically force mercury—a common liquid metal—into pores that it otherwise would not naturally fill.

Thus, it is remarkably interesting that Prof. Xiaolin Wang and colleagues were able to coerce liquid metal (gallium-based liquid metals) to pass through paper under the influence of gravity [1]. Figure 1 (adapted from Fig. 2 from [1]) shows examples of liquid metal passing through the pores of paper and other porous materials. The metal is able to pass through the pores because of electrochemical oxidation of the metal surface, achieved by applying a positive potential to the metal relative to a counter electrode in an aqueous solution of NaOH. The potential drives the formation of oxide species on the metal and thereby lowers the tension of the metal. Normally,

potentials can lower the tension of metals via electrocapillarity, which modulates the amount of charge at the surface of the metal (like a capacitor). However, elec-

trocapillarity cannot explain the behavior observed here because the changes in tension are much larger than would be possible by electrohydrodynamics [2].

**Figure 1.**
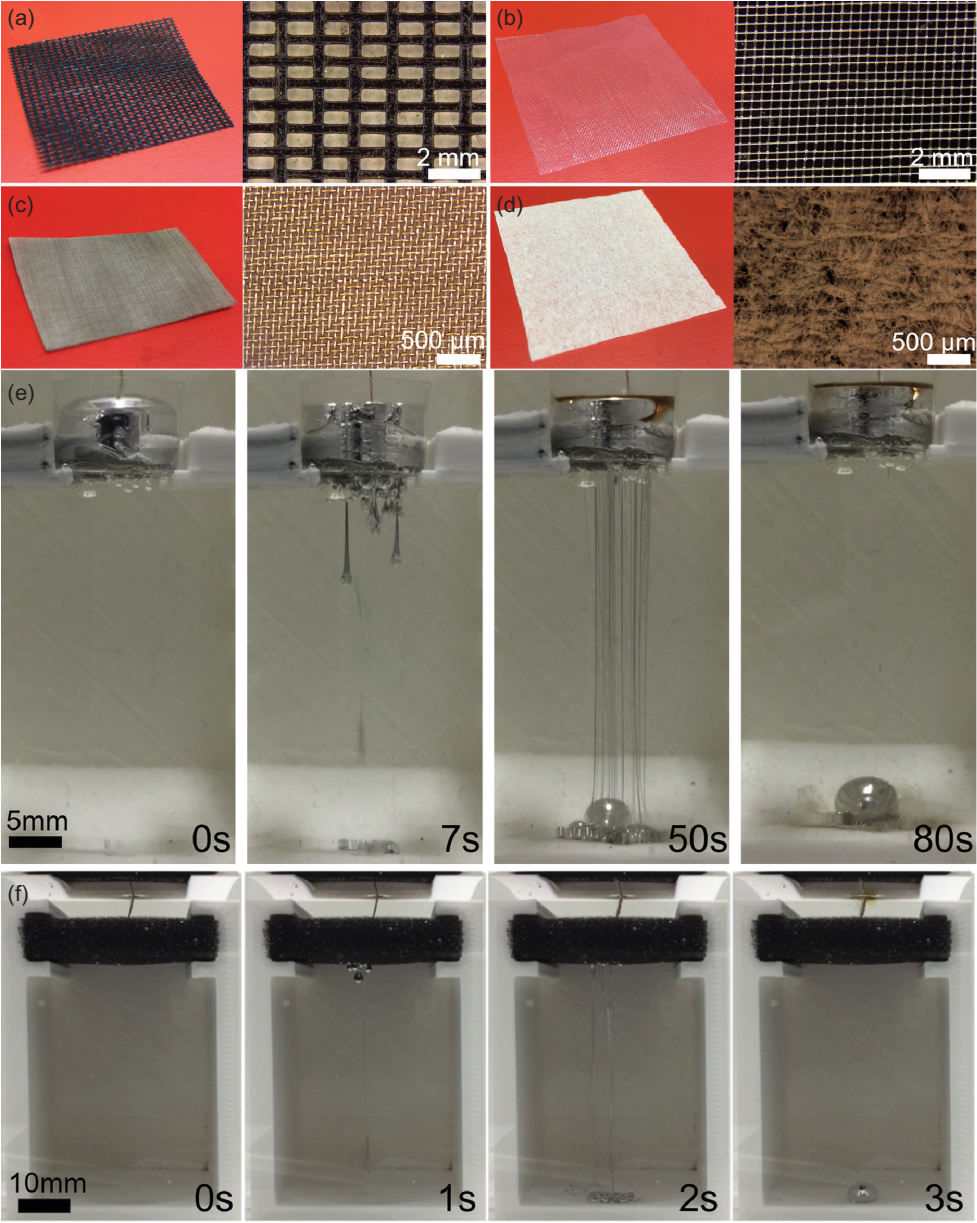
Porous media (a–d) normally prevent liquid metals (shown in the left-most column of (e) and (f)) from passing through the pores because of the high tension of the metal. However, applying an oxidizing potential to the metal drives surface oxidation that lowers the tension, thereby allowing gravity to pull the metal through the pores (adapted from [1]).

Electrochemical oxidation creates a surface oxide that lowers the tension [3] in a manner similar to a surfactant. In addition, there is evidence to suggest that current (ions) driven through the oxide generates compressive forces on the surface [4]. Liquids usually form shapes that minimize their surface energy (hemi-spherical droplets); thus, the observation that the metal can form fractals (observed previously under similar conditions [4]) or fibers (as seen in [1]) suggests the tension is near zero and thus, there is no energetic penalty for the metal to increase its area under the influence of gravity.

The ability to manipulate the shape of liquid metals has implications for actuators and reconfigurable electronic, optical and thermal structures that rely on the properties of metals. Beyond applications, this work is simply ‘cool’ because the results are so striking and defy conventional expectations.

## References

[1] Yun FF , Yu ZW, He YH et al. Natl Sci Rev 2020; 7: 366–72. 10.1093/nsr/nwz168 PMC8288959 34692052

[2] Eaker CB , Dickey MD. Appl Phys Rev 2016; 3: 031103.

[3] Khan MR , Eaker CB, Bowden EF et al. Proc Natl Acad Sci USA 2014; 111: 14047–51. 25228767 10.1073/pnas.1412227111 PMC4191764

[4] Eaker CB , Hight DC, O’Regan JD et al. Phys Rev Lett 2017; 119: 174502. 29219460 10.1103/PhysRevLett.119.174502

